# Supplementary material for: City-Level Travel Time and Individual Dietary Consumption in Latin American Cities: Results from the SALURBAL Study
Source: Int J Environ Res Public Health. 2022 Oct 18;19(20):13443. doi: 10.3390/ijerph192013443 (PMC9602577; doi:10.3390/ijerph192013443)
Supplement: Supplementary file 1 [file ijerph-19-13443-s001.zip › ijerph-1948108-supplementary.pdf]

Supplementary Materials S1: Questions and answers (translated to English) used to create days per week with vegetable consumption in the national health surveys, 2010–2016.

- **Chile (Encuesta Nacional de Salud—ENS2010)* and Peru (Encuesta Nacional de Demografia y Salud—ENDES2016)****

  Question: "In a typical week, how many days do you eat vegetables?"

  Response options: Number of days (continuous) ranging 0–7; or Don't know/refused.

  * Specifically asks to not include potatoes and legumes.

  ** Specifically asks only "vegetable salads".

- **Brazil (Pesquisa Nacional de Saúde—PNS 2013)**

  Question 1: "On how many days of the week do you usually eat salad or raw vegetables?"

  Response options: Never or less than once per week; Number of days (continuous) ranging 1–7; or Don't know/refused.

  Question 2: "On how many days of the week do you usually eat cooked vegetables?"

  Response options: Never or less than once per week; Number of days (continuous) ranging 1–7; or Don't know/refused.

- **Colombia (Encuesta Nacional de la Situation Nutricional en Colombia—ENSIN2010)**

  Question 1: "Usually in the last month do you eat cooked vegetables?"

  Response Options: Yes—Go to Question 2; or No—Go to Question 3

  Question 2: "How often do you eat cooked vegetables?"

  Response Options: Less than once per month; Once a month; Two or three times per month; Once a week; Twice a week; Three or four times per week; Five or six times per week; Once a day; Twice a day; or Three times or more per day.

  Question 3: "Usually in the last month do you eat raw vegetables?"

  Response Options: Yes—Go to Question 4; No—End.

  Question 4: "How often do you eat raw vegetables?"

  Response options: Less than once per month; Once a month; Two or three times per month; Once a week; Twice a week; Three or four times per week; Five and six times per week; Once a day; Twice a day; or Three times or more per day.

- **Mexico (Encuesta Nacional de Salud y Nutrición—ENSANUT2012)**

  Question (assessed using a Food frequency questionnaire): "How many days did you eat (type of vegetable)?" This question is repeated for each type of vegetable.

  Response options: Never; 1 day; 2–4 days; 5–6 days; or 7 days.

Supplementary Materials S2. Questions and answers (translated to English) used to create days per week with sugar-sweetened beverages consumption in the national health surveys, 2010–2016.

- **Brazil (Pesquisa Nacional de Saúde—PNS 2013)**

  Question 1: "How many days per week do you usually drink soda (or artificial juice)?"

  Response options: Never or less than once per week; or Number of days ranging 1–7.

  Question 2: "What type of soda or juice do you usually drink?"

  Response options: Normal/regular; Diet/light/zero; or Both.

- **Colombia (Encuesta Nacional de la Situation Nutricional en Colombia—ENSIN2010)**

  Question 1: "Usually in the last month do you consume soda or drinks (powder, in a box, or bottle)?"

  Response Options: Yes—Go to Question 2; No—End.

  Question 2: "How often do you consume soda?"

  Response Options: Less than once per month; Once a month; Two or three times per month; Once a week; Twice a week; Three or four times per week; Five or six times per week; Once a day; Twice a day; Three times or more per day.

- **Mexico (Encuesta Nacional de Salud y Nutrición—ENSANUT2012)**

  Question (assessed using a Food frequency questionnaire): "How many days did you consume (type of SSB)?" This question is repeated for each type of SSB. The SSBs included for calculation of days per week are: 1) regular soda; 2) fruit water with sugar; 3) industrialized drinks or flavored waters with sugar; 4) fruit nectars or industrialized fruit pulp with sugar. Additional beverages included for type of SSB usually consumed are 5) diet soda; 6) industrialized drinks or flavored waters without sugar.

  Response options: Never; 1 day; 2–4 days; 5–6 days; or 7 days.
